# Usage Patterns of Cosmetic and Personal Care Products among Female Population in Saudi Arabia: Important Factors for Exposure and Risk Assessment

**DOI:** 10.1155/2020/8434508

**Published:** 2020-04-08

**Authors:** Heba Shaaban, Wejdan Alhajri

**Affiliations:** Department of Pharmaceutical Chemistry, College of Clinical Pharmacy, Imam Abdulrahman Bin Faisal University, King Faisal Road, P.O. Box 1982, Dammam 31441, Saudi Arabia

## Abstract

Reliable data regarding the usage patterns of personal care products (PCPs) are needed to determine the health risks posed by the ingredients of these products such as parabens, phthalates, and bisphenol A. There are no published data regarding the consumption patterns of PCPs in the Middle East in general and in Saudi Arabia in particular. To fill this gap, this study aimed to assess important factors such as the percentage of users and the frequency of use and co-use of twenty-three cosmetic and PCPs among the female population in Saudi Arabia. Additionally, this study aimed to assess the common cosmetic-related adverse events among the participants. The studied products included general hygiene, hair care, skin care, makeup, fragrances, and other products. The data were collected from 709 female participants of different age groups through a digital questionnaire. It was found that eighteen of the investigated products are consumed by at least 50% of the respondents. The frequency of use of PCPs varied over a wide range. Cosmetic-related adverse events were experienced by 16.1% of the participants. Use frequencies of many hygiene and makeup products were correlated with each other. This study provides, for the first time, baseline data on the usage patterns of a large number of widely consumed PCPs among female population pertaining to several sociodemographic strata. Such information is crucial for exposure and risk assessment and also needed for updating the current knowledge on usage of PCPs in Saudi Arabia.

## 1. Introduction

Personal care products (PCPs) are widely consumed by people of all ages. These products can be used on a daily basis such as deodorants, facial moisturizers, or creams [1]. Some chemicals are used in the manufacturing of PCPs for various purposes. For example, parabens are used as preservatives because of their antimicrobial activity and phthalates are used as solvents and fixative in fragrances [2]. Parabens are endocrine disruptors that have weak estrogenic activity in some in vitro screening tests, such as ligand binding to the estrogen receptors and proliferation of MCF-7 cells [3]. The negative impact of these harmful chemicals contained within cosmetics and PCPs is not confined only to humans as it can also affect the environment and animals [4, 5]. Other chemicals can also be found in PCPs such as triclosan, heavy metals, hydroquinone, and nitrosamines [6]. Occurrence of these compounds in PCPs may cause negative health impacts including allergy, endocrine disruption, birth defects, neurotoxicity, or cancers [6, 7].

The progress in the cosmetic industry and the emergence of a large number of manufactured products in the last century has resulted in an increase of PCP consumption, leading to excessive exposure of the general population to a wide variety of chemicals that may pose adverse health effects [8, 9]. Because parabens and phthalates are the most concerned harmful chemicals found in PCPs, various analytical methods have been developed to evaluate the content of these chemicals in different samples [10–14].

In order to assess health risks for consumers and to determine the exposure risk of endocrine disrupting compounds from consuming PCPs, important predictors such as use prevalence, use frequency, and co-use pattern of these products should be available [15]. A series of studies have been conducted in different countries to assess the consumption pattern of PCPs in different populations. For example, Biesterbos et al. evaluated the use frequency of thirty-two cosmetics including general hygiene, skin care, hair care, and makeup products in the Netherlands [16]. This study showed that the majority of participants (>50%) indicated to use two or more products simultaneously and the co-use of the products was highly observed with nail polish and nail polish remover [16]. Manova et al. assessed the frequency of use of eight leave-on care products by Swiss populations [17]. This study revealed that the majority of respondents (99%) reported having used at least one of the investigated products in the past year and the co-use of products was common and more complex among adults [17]. Additionally, Wu et al. determined the usage patterns of thirty PCPs among 604 California households [1]. Furthermore, a study was conducted in France by Ficheux et al. to assess the consumption pattern of 106 products among French consumers [18]. This study demonstrated that adult women used an average of 27 cosmetics per year versus 12 for adult men. Another study was also conducted in France and focused on the assessment of usage pattern of hair care products among French women [19].

To the best of our knowledge, no published data regarding the usage patterns of PCPs and their adverse events among female population in Saudi Arabia are available and because of the importance of this information to assess exposure and risk assessment, it was decided to conduct this study.

The use of nonmedicated cosmetics is very common among Saudi Arabian female population and little is known about the product-related adverse events, and thus the findings of this study will provide baseline important information regarding important predictors such as percentage of users, frequency of use, co-use pattern of PCPs, and their adverse events among females in Saudi Arabia. Such information is of a paramount interest to public health as it is possible that the same chemical could be found in many products used by a consumer on a daily basis leading to adverse health effects. Additionally, this study aimed to draw the attention of consumers who are more vulnerable to the adverse effects of the chemicals contained in PCPs. The data presented in this study could provide beneficial input for future exposure and risk assessment modeling.

## 2. Materials and Methods

### 2.1. Study Population

A cross-sectional, survey-based study was conducted among females in the Eastern Province of Saudi Arabia between September 2018 and December 2018. The online survey was used as a tool for data collection. All the inclusion criteria, i.e., being a female, are outlined in the participant information sheet attached to the survey. This study considered female population because they are more likely to use cosmetic and PCPs, and thus they are more vulnerable to adverse effects of these products. The study included participants aged from 18 to 70+ years. Including several age groups allowed us to evaluate the variability of consumption patterns across age.

### 2.2. Sample Size

The sample size was calculated online using an online sample calculator (Raosoft). Based on the statistical data and official figures released by the Central Department of Statistics and Information, the number of females in the Eastern Province of the country was ∼1,506,116 as of December 2017. Therefore, 385 participants were needed to obtain a 95% confidence interval with a 5% margin of error. After exclusion of incomplete and contradictory responses, the final data included 709 participants.

### 2.3. Study Instrument

For developing the questionnaire, a literature review was undertaken. The questionnaire was conducted in both Arabic and English languages. The first draft of the questionnaire was presented to a panel of experts including two college professors who are professionally trained and familiar with the concepts being examined in the study for their opinion and comments on the contents of the questionnaire. Based on their recommendations, necessary omission, addition, and language editing were performed to ensure participant understanding. Then, the final version of the questionnaire was posted online.

The survey consists of two sections, and only closed-end questions were used with the questionnaire specifying possible answers. These questions have the advantages of being quick to administer, easy to answer, and easier to analyze and interpret than open questions [20]. The first section of the questionnaire was mainly focused on demographics of participants such as age, education level, and nationality. The second section was designed to estimate the use-patterns of twenty-three cosmetic and PCPs including general hygiene, skin care, hair care, makeup, fragrances, and tanning products (see Table 1). Respondents were asked to check all the products they had used at least once during the past 6 months and to indicate the corresponding frequency of use.

### 2.4. Data Analysis

The collected data were checked to exclude any error or inconsistency. The responses were analyzed using SPSS (Statistical Package for Social Sciences) (IBM Corp., Armonk, NY, USA) version 22.0 and Excel program. The descriptive statistics were generated, and descriptive data in terms of percentage and frequency were used to demonstrate the findings. The chi-square test was used to assess possible relationships between different variables. For all statistical tests, a *p* value <0.05 was considered significant. Spearman correlation coefficient (*R*) was used to measure the strength of correlations between the use of different products. Correlation was considered weak if 0.2 < *R* > 0.39, moderate if 0.4 < *R* > 0.59, strong if 0.6 < *R* > 0.79, and very strong if 0.8 < *R* > 1.00 (*p* values <0.05).

## 3. Results

### 3.1. Participants' Characteristics

In total, 709 participants completed the questionnaire. For data analysis, two age groups were constructed, young females (18–39 years) and senior females (40–70+ years). These groups contained 82.2% and 17.8% of the respondents, respectively. The respondents were also divided into two groups based on the level of education: low (primary, elementary, or high school) and high (with a college degree). Most of the respondents (81%) are of a higher education level. Of the respondents, 89.1% were Saudis. The detailed demographics of the participants are shown in Table 2.

### 3.2. Prevalence of Use

**Figure 1:**
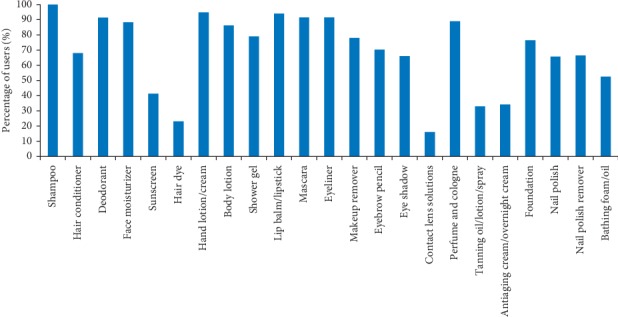
General overview of the percentages of users by product for all respondents (*N* = 709).

The prevalence of use was defined as the percentages of users who reported the use of a PCP at least once in the past 6 months. The results indicated that all the respondents reported the use of at least one of the investigated products. The proportion of the users varied among the investigated products. The results showed that 18 of the investigated PCPs are consumed by at least 50% of the respondents. Of the hair care products, shampoo was used by all the participants (100%), while conditioner was used by 68% of the participants. The majority of respondents used hand lotion/cream (95%) and deodorant (91%). The use of makeup products is highly prevalent among the respondents. The special care products such as contact lens solutions were used by only 16% of the participants.  Figure 1 shows a general overview of the percentage of users per product for all respondents (*N* = 709).

Among young female users (18–39 years), the prevalence of use was higher for nail polish and nail polish remover (*p* ˂0.05). On the other hand, senior female users reported a higher prevalence of using hair dye, antiaging cream, and tanning products (*p* < 0.05). With respect to the education level, there was no significant difference observed in the prevalence of use for all products except for nail polish. More users with a college degree used nail polish (67.1%) compared to users without a college degree (60.7%, *p* < 0.05). Unfortunately, the sample size of some age and education-level groups did not allow further comparisons.

### 3.3. Frequency of Use

**Figure 2:**
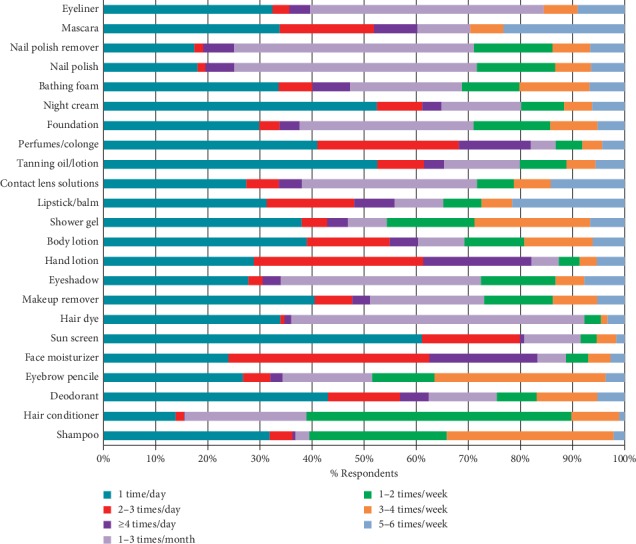
Frequency of use among respondents (*n* = 709) for the PCPs studied.

It was observed that some cosmetic products are used on a daily basis by consumers. The majority of users (˃50%) reported the use of deodorant, sunscreen, and night cream once a day, and 40% of the users indicated to use lipstick/balm, shower gel, body lotion, makeup remover, and eyebrow pencils one time per day. Hair conditioner was used once or twice per week by 82% of the users. Nail polish/nail polish remover was mostly used one to three times per month by more than 46%. Conversely, hair dyes and contact lens solutions are less frequently used. The young age group used hair dye less frequently compared to the senior age group. The frequency of use of the other products was much more diverse, and a predominant frequency of use could not be assessed. The frequency of use of the products is summarized in Figure 2.

### 3.4. Co-Use of Different Products

The data regarding the co-use of products are important to assess the magnitude of exposure to different products that contain the same chemicals. Co-use data are important also for risk assessment. Therefore, we examined the correlations of use for the studied products. It was observed that the use of many hygiene products was correlated with each other, e.g., the use of shower gel, shampoo, body lotion, and shower gel was moderately correlated (*R* ranged from 0.409 to 0.428). Also, moderate correlations were found between different types of makeup products, e.g., makeup remover, foundation, eye shadow, eye pencils, and mascara (*R* ranged from 0.403 to 0.60). Additionally, a very strong correlation was found between the use of nail polish and nail polish remover (*R* is 0.977) and also between the use of tanning oil and antiaging creams (*R* is 0.976). Such a pattern of continuous co-use of products can increase the exposure to endocrine disrupting compounds contained in these products leading to harmful effects. The co-use data of the investigated products are presented in Table 1, showing the spearman values (*R*).

### 3.5. Cosmetic Product-Related Adverse Events

**Figure 3:**
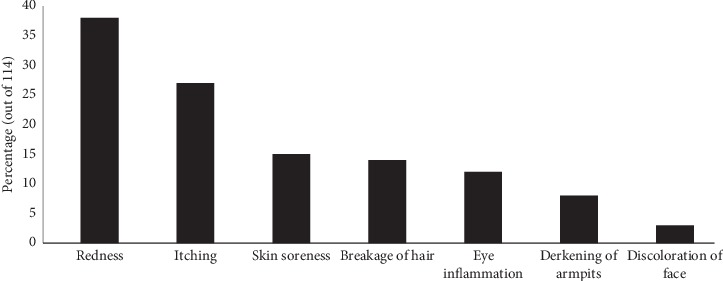
Reported cosmetic-related adverse events.

It was found that 114 (16.1%) of the participants had experienced adverse effects from the use of cosmetic products. The most complained cosmetic products were lotions (51.2%), face creams (27.1%), and deodorants (10.3%). The most reported adverse events were redness, itching, skin soreness, breakage of hair, eye inflammation, darkening of armpits, and discoloration of face (see Figure 3).

## 4. Discussion

In this study, we created a database containing specific information regarding the current usage patterns of twenty-three PCPs. This database includes the prevalence of use, frequency of use, and the co-use of PCPs. To the best of our knowledge, no similar studies that include the usage patterns of PCPs among female population in Saudi Arabia have been published previously; therefore, the results of this study was compared with the studies conducted outside Saudi Arabia. As frequency of use data is required for assessing exposure to ingredients in PCPs, this study can provide essential information for exposure and risk estimation.

In general, we found that the majority of respondents were more likely to use many of the investigated products such as hygiene products and makeup products and the frequency of use of the PCPs studied varied widely. This variation was also observed by [1]. The wide use of PCPs observed in this study may be attributed to the progress in the cosmetic industry and the expandable cosmetic companies advertising for cosmetic and PCPs.

The prevalence of use for some products varied by age, for example, senior females were heavier users of hair dye and antiaging creams than younger females. This finding is expected as the use of some products may not be common among younger users (e.g., antiaging products). On the other hand, younger females are more likely to use nail polish and nail polish remover than others. Our results were in good agreement with the study conducted by Biesterbos et al. [16] who observed that the usage patterns are varied by age. The exposure information based on age could be beneficial when designing future intervention to raise the awareness or draw the attention of a specific population. It was found that the educational level has no influence on participants' choices of products. This finding contradicted the study conducted by Wu et al. [1] who demonstrated that education is an important factor influencing the consumption of some products; for example, highly educated participants are heavier users for sunscreen than others.

The importance of investigating use frequency is to provide information beneficial for estimating aggregate and cumulative exposure to ingredients with negative health impacts contained in PCPs. The simultaneous use of products containing the same ingredients would lead to aggregate exposure [7]; therefore, it was necessary to investigate the use of PCPs from the same class.

Regarding the self-reported adverse events, 16.1% of the participants experienced adverse events. Complaints against the adverse effects caused by cosmetics were in concurrence with other studies [21–24]. Lotions and face creams were the most cosmetic products reported to cause adverse effects, followed by deodorants. Similarly, many population-based studies reported that the top complained cosmetics were deodorants and lotions, e.g., [22, 23].

It was observed that there is a correlation between the use of products within the same class and also from other classes. For example, a correlation was found between the use of many hygiene products such as shower gel, shampoo, body lotion, and shower gel. However, there was weak association between the use of shampoo and hair conditioner. This observation is similar to the study conducted by Biesterbos et al. who reported that there was no correlation between the use of shampoo and hair conditioner [16]. Also, moderate correlations were found between different types of makeup products such as makeup remover, foundation, eye shadow, eye pencils, and mascara. The correlation between the use of makeup remover and other makeup products may be attributed to the consumers' awareness of the importance of removing the makeup after use. Additionally, a very strong correlation was found between the use of nail polish and nail polish remover. This finding is also in an agreement with the studies conducted by Wu et al. and Biesterbos et al. [1, 16].

## 5. Strengths and Limitations

The strength of this study is the consideration of a large number of cosmetic and PCPs among a priori at-risk subpopulation. The study highlighted an area where very little research has been conducted. The provided information can act as a guide in the next step of consumer exposure modeling of PCPs as well as aggregate exposure assessment for substances contained in PCPs.

One of the limitations of this study is that despite the large sample size of 709 participants which consisted of female from different age groups and educational level, not equal proportion of highly educated and lower educated participants were gathered, which hindered a fair comparison. Also, in this study, a predefined assumption that only females are more likely to use cosmetic products has been made, and the study was confined to investigate the usage pattern among females only. Such an assumption can cause information to be missed, as men may use cosmetics professionally (e.g., actors) and male cosmetic use is also becoming more accepted in some societies. Therefore, it is very interesting to investigate the consumption pattern of cosmetic and PCPs among men as well. Assessment of the usage pattern based on gender could give insights into the usage pattern for the whole population.

## 6. Recommendations

- The findings of this study emphasize the need for future wellbeing educational programs to raise public awareness regarding the health risks posed by the chemicals contained in personal care and cosmetic products, especially among adolescents and young adults in Saudi Arabia.
- A significant role can also be played by health care providers who are at the forefront in guiding and providing proper education and awareness to the community regarding the health impact of prolonged exposure and consumption of PCPs.
- It is also important to ensure the quality of cosmetic and PCPs marketed in Saudi Arabia in order to assure the consumers' safety. This study could open the door for future studies aiming at assessing the exposure risks associated with consumption of PCPs among Saudi population.
- It is worthy to mention that this study was conducted at a specific point of time (when the study was performed), and because participants' choices may undergo changes over time according to the shifts in cosmetic industry and the rise in the public health awareness regarding the use of PCPs, repeated assessment may enable tracking the consumers' usage pattern over time.

## 7. Conclusion

This study provides recent information on the individual usage patterns of a large number of widely consumed PCPs by female population in Saudi Arabia. It was found that the consumption of PCPs varies widely and different products are consumed simultaneously. Reporting adverse effects of cosmetic products necessitates the consideration of safety concerns related to cosmetic use. Promoting the concept of cosmetovigilance among cosmetic users, sellers, and other stakeholders should be taken into consideration. The findings of this study could be beneficial for safety assessors in order to protect the general population and those who are at risk. Current frequency use and co-use data are crucial to determine realistic exposure to cosmetics and PCPs by the Saudi population.

## Figures and Tables

**Table 1:** Correlation for use of different cosmetic products among study subjects.

Product	Deodorant	Shampoo	Hair conditioner	Face moisturizer	Sunscreen	Hair dye	Hand lotion	Body lotion	Shower gel	Lipstick	Mascara	Eyeliner	Makeup remover	Eyebrow pencil	Eye shadow	Contact lens solutions	Perfumes	Tanning oil	Anti-aging creams	Foundation	Nail polish	Nail polish remover	Bathing foam/oil
Shampoo	0.272	—	0.361	0.160	0.077	0.099	0.111	0.272	0.248	0.178	0.248	0.160	0.206	0.160	0.162	—	0.132	0.172	0.168	0.181	0.100	0.107	0.231
Hair conditioner	0.155	0.361	—	0.146	0.156	0.251	—	0.181	0.298	0.092	0.140	0.174	0.216	0.174	0.208	—	0.168	0.242	0.219	0.213	—	0.088	0.265
Deodorant	—	0.272	0.155	0.189	0.140	—	0.112	0.223	0.266	0.166	0.138	0.111	0.162	0.111	—	0.135	—	0.088	0.097	0.104	—	—	0.152
Face moisturizer	0.189	0.160	0.146	—	0.224	—	0.392	0.347	0.186	0.310	0.222	0.204	0.207	0.204	0.139	0.149	—	0.143	0.151	0.156	0.151	0.147	0.113
Sunscreen	—	0.077	0.156	0.224	—	0.227	0.120	0.185	0.213	0.156	0.143	0.170	0.215	0.170	0.192	—	0.232	0.386	0.404	0.190	0.191	0.188	0.212
Hair dye	—	0.099	—	0.042	—	—	—	—	0.098	—	0.091	0.130	0.112	0.130	0.167	—	0.359	0.263	0.276	0.202	0.119	0.112	0.202
Hand lotion/cream	0.112	0.111	0.029	0.392	0.120	—	—	0.372	0.191	0.324	0.212	0.134	0.165	0.134	0.084	0.209	—	0.089	0.077	0.121	—	—	0.128
Body lotion	0.223	0.272	0.181	0.347	0.185	—	0.372	—	0.416	0.245	0.258	0.188	0.289	0.188	0.174	0.142	0.094	0.146	0.146	0.194	0.156	0.159	0.272
Shower gel	0.266	—	0.298	0.186	0.213	0.098	0.191	0.416	—	0.202	0.226	0.200	0.251	0.200	0.180	0.139	0.146	0.237	0.220	0.189	0.144	0.140	0.409
Lipstick/lip balm	0.166	0.178	0.092	0.310	0.156	—	0.343	0.245	0.202	—	0.403	0.276	0.303	0.276	0.115	0.243	—	—	—	0.195	0.154	0.147	0.161
Mascara	0.138	0.248	0.140	0.222	0.143	—	0.212	0.258	0.226	0.403	—	0.467	0.610	0.467	0.390	0.161	0.083	0.108	0.120	0.460	0.247	0.241	0.185
Eyeliner	0.111	0.160	0.174	0.204	0.170	0.130	0.134	0.188	0.200	0.276	0.467	—	0.488	0.462	0.433	—	0.235	0.215	0.225	0.364	0.264	0.293	0.170
Makeup remover	0.162	0.206	0.216	0.207	0.215	0.112	0.165	0.289	0.251	0.303	0.610	0.488	—	0.488	0.441	0.189	0.178	0.225	0.235	0.515	0.347	0.340	0.271
Eyebrow pencil	0.111	0.160	0.174	0.204	0.170	0.130	0.134	0.188	0.200	0.276	0.467	0.462	0.488	—	0.433	—	0.235	0.215	0.225	0.364	0.264	0.263	0.170
Eye shadow	—	0.162	0.208	0.139	0.192	0.167	0.084	0.174	0.180	0.115	0.390	0.433	0.441	0.433	—	0.046	0.221	0.225	0.230	0.473	0.391	0.382	0.232
Contact lenses	0.135	—	—	0.149	0.023	—	0.209	0.142	0.139	0.243	0.161	0.133	0.189	0.133	0.046	—	—	0.105	0.090	—	0.089	0.080	0.133
Perfume	—	0.132	0.168	0.048	—	—	—	0.094	0.146	0.041	0.083	0.235	0.178	0.235	0.221	—	—	0.301	0.353	0.169	0.171	0.170	0.233
Tanning oil/lotion	0.088	0.172	0.242	0.143	—	0.263	0.089	0.146	0.237	—	0.108	0.215	0.225	0.215	0.226	0.105	0.301	—	0.977	0.220	0.112	0.109	0.277
Antiaging creams	0.097	0.168	0.219	0.151	—	—	—	0.146	0.220	—	0.102	0.225	0.235	0.225	0.230	0.090	0.353	0.977	—	0.220	0.115	0.114	0.286
Foundation	0.104	0.181	0.213	0.156	0.190	0.202	0.121	0.194	0.189	0.195	0.460	0.364	0.515	0.364	0.473	0.066	0.169	0.220	0.220	—	0.403	0.394	0.240
Nail polish	—	0.100	0.080	0.151	0.190	0.119	—	0.156	0.144	0.154	0.240	0.264	0.347	0.264	0.391	0.089	0.171	0.112	0.115	0.403	—	0.970	0.264
Nail polish remover	—	0.107	0.088	0.147	0.188	0.112	—	0.154	0.140	0.147	0.420	0.263	0.340	0.263	0.382	0.080	0.170	0.109	0.114	0.394	0.970	—	0.259
Bathing foam/oil	0.152	0.231	0.265	0.113	0.212	0.202	0.128	0.272	0.409	0.161	0.100	0.170	0.271	0.170	0.232	0.133	0.233	0.277	0.286	0.240	0.264	0.259	---

Spearman correlation coefficients were used. Only correlation with *p* < 0.05 is shown.

**Table 2:** Demographic characteristics of the participants (*n* = 709).

Variables		Numbers (*n*)	Percentage
Age	18–39	583	82.2
	40–70+	126	17.8

Education	Primary/elementary school	27	3.8
	High school	108	15.2
	College/university degree	574	81

Living region	Eastern region	464	65.4
	Middle region	121	17.1
	Western region	64	9.0
	Southern region	32	4.5
	Northern region	28	3.9

Nationality	Saudi	632	89.1
	Gulf	24	3.4
	Arab-Asian	30	4.2
	Arab-African	14	2.0
	Others	9	1.3

## Data Availability

The data used to support the findings of this study are available from the corresponding author upon request.
